# Localization of fat depots and cardiovascular risk

**DOI:** 10.1186/s12944-018-0856-8

**Published:** 2018-09-15

**Authors:** Olga Gruzdeva, Daria Borodkina, Evgenya Uchasova, Yulia Dyleva, Olga Barbarash

**Affiliations:** 1 Federal State Budgetary Institution, Research Institute for Complex Issues of Cardiovascular Diseases, Kemerovo, Russian Federation; 2 Federal State Budget Educational Institution of Higher Education, Kemerovo State Medical University of the Ministry of Healthcare of the Russian Federation, Kemerovo, Russian Federation; 3 Autonomous Public Healthcare Institution of the Kemrovo Region, Kemerovo Regional Clinical Hospital named after S.V. Beliyaev, Regional Center for Diabetes, Kemerovo, Russian Federation

**Keywords:** Visceral obesity, Epicardial adipose tissue, Perivascular adipose tissue, Non-alcoholic fatty liver disease, Cardiovascular disease

## Abstract

Despite the existing preventative and therapeutic measures, cardiovascular diseases remain the main cause of temporary disability, long-term disability, and mortality. Obesity is a major risk factor for cardiovascular diseases and their complications. However, not all fat depots have the same inflammatory, paracrine, and metabolic activities. In addition, recent studies have indicated that the accumulation of visceral fat, rather than subcutaneous fat, is associated with increased cardiometabolic risk. However, there is also evidence that increasing the area of visceral fat can help protect against lipotoxicity. This review aims to discuss the contemporary literature regarding the characteristics of the visceral, epicardial, and perivascular fat depots, as well as their associations with cardiovascular disease.

## Background

Despite the existing preventative and therapeutic measures, cardiovascular diseases (CVD) remain the main cause of temporary disability and mortality [1]. Furthermore, CVD caused 17.3 million deaths in 2013, which is 40.8% higher than the figure from 1990 [2]. This increase is partially related to population growth and aging, although cardiovascular risk factors continue to play a non-neglible role. Many epidemiological and clinical studies have significantly expanded our understanding of unmodifiable and modifiable risk factors [3, 4], with the development of CVD and related mortality being associated with a high body mass index (BMI), arterial hypertension, and increasing concentrations of glucose and cholesterol [5]. During the last 50 years, measures that reduced the prevalence of smoking, hypertension, and hypercholesterolemia have reduced the coronary artery disease (CAD) mortality rate by approximately 2-fold in economically developed countries [6]. Nevertheless, the current consumption of high-calorie foods and decreasing activity have made obesity and type 2 diabetes mellitus the leading risk factors for CAD progression and mortality [7]. Moreover, the large Framingham and the Nurses’ Health Studies have demonstrated that obese patients have a 2-fold higher risk of heart failure and a 4.1-fold higher risk of CVD progression relative to patients with normal weight [8, 9]. Therefore, this review aims to discuss the contemporary literature regarding the characteristics of various fat depots and their associations with cardiovascular disease.

### Regional body fat distribution and cardiovascular/metabolic risks

Obesity reflects an excess of adipose tissue and is traditionally estimated using BMI, which is an anthropometric measure based on the patient’s weight and height (kg/m^2^) [10]. During the last 10 years, many promising studies have revealed a U-shaped relationship between BMI and CVD mortality, with the relationship being observed in all ethnic groups and not being dependent on sex [11, 12]. In addition, studies from the early 1980s in Sweden and the US convincingly demonstrated that a simple anthropometric method for evaluating the regional distribution of adipose tissue (the ratio of waist circumference to hip circumference) was more effective than BMI for assessing the risk of metabolic and cardiovascular complications [13].

A number of studies have examined the topic of metabolic syndrome (MS), which includes hypertension, hyperglycaemia, and dyslipidaemia in the absence of obesity, and their results led to the proposal of the term “metabolically healthy obesity” [14]. This term describes the marked metabolic heterogeneity of obesity, which is related to the distribution of fat in different ectopic depots, and highlights the importance of a more nuanced approach to assessing CVD risk. The usual methods for visualising adipose tissue include computed tomography (CT), magnetic resonance imaging, and ultrasonography, which can detect each patient’s fat distribution. Thus, ectopic fat depots are divided into two subtypes that have predominantly systemic effects (visceral adipose tissue and fat deposits in the liver and skeletal muscles) or predominantly local effects (perivascular, epicardial, and perivascular fat depots) [15]. Most ectopic fat deposits are closely associated with cardiometabolic risks and the clinical manifestations of most CVDs [16, 17] (Table 1).

**Table 1:** The relationship between various depots of fat and cardiovascular diseases

Feature	SAT	Adipose tissue around the visceral organs				Ectopic fat	
		VAT	EAT	PVAT	PNAT	Liver	Muscles
Location	Between the dermis and fascia	Adipose tissue around the abdominal visceral organs	Visceral fat between the myocardial surface and the visceral layer of the pericardium	Adipose tissue around the vessels (coronary arteries) regardless of location	Fat around the kidneys	Lipid (triglycerides) deposits in non-adipose liver tissue	Lipid (triglycerides) deposits in non-adipose muscle tissue
Type of adipose tissue	White	White	White	Brown/white	Brown	–	–
Imaging Modality							
Ultrasonography	–	−/+	+	–	–	+	–
Multidetector computed tomography	+	+	+	+	+	+	–
Magnetic resonance imaging	+	+	+	+	+	+	+
Magnetic resonance spectroscopy	–	–	–	–	–	+	+
Factors expressed							
Peroxisomal proliferator	+	++		–		–	–
Activated receptor-γ (PPAR-γ)	++	+ (obesity)		+/−		–	–
Uncoupling protein-1	–	–	–	+	+	–	–
Uncoupling protein-2	+	++	+	–		–	–
Secretory activity							
Lipoprotein lipase	+	+		–	+	+	+
Free fatty acids	+	++ (morbid obesity)	++	+	+	VLDL	+
Plasminogen activator inhibitor-1	++	+	+	++ (morbid obesity)	+	–	–
Leptin	+	++	+	++ (morbid obesity)	+	–	–
Resistin			–	++ (morbid obesity)	–	–	–
Angiotensinogen	++	+	–	+	+	–	–
Adiponectin	++	+	–	+		–	–
Tumor necrosis factor-α	+	+	+	–	+	–	–
Interleukin-6	++	+	+	++ (morbid obesity)	+	–	–
Interleukin-1β	+	+	+	++ (morbid obesity)	+	–	–
Effects	Protective production of adiponectin	• Gluconeogenesis • Insulin resistance • Dyslipidemia • Systemic inflammation	• Protecting cardiomyocytes from hyperthermia • Synthesis of adiponectin and adrenomedullin • Source of energy during ischemia • Absorption of excess FFA • Synthesis of inflammation markers • Associated with myocardial hypertrophy • Myocardial fibrosis and apoptosis of cardiomyocytes	Vasodilation	• Associated with microalbuminuria • Thermoregulation • Structural involvement in the regulation of renal vessel tone • Synthesis of inflammation markers	• Gluconeogenesis • Insulin resistance • Dyslipidemia	• Insulin resistance • Dyslipidemia

*SAT* subcutaneous adipose tissue, *VAT* visceral adipose tissue, *EAT* epicardial adipose tissue, *PVAT* perivascular adipose tissue, *PNAT* paranepalic adipose tissue

### Visceral adipose tissue

In the 1980s, Fujioka et al. and Sjöström et al. demonstrated that fat tissue distribution does not depend on BMI, [18, 19] although the accumulation of total body fat is related to fat deposition in the subcutaneous and visceral depots. During the initial stage, the deposition predominantly involves the subcutaneous depot, [20] with a gradual and disproportionate transition towards adipose tissue deposition in the visceral depots. The development of abdominal-visceral obesity is combined with unfavourable metabolic activity and an increased risk of cardiovascular complications. In this context, the metabolic activity of visceral fat is considered a key factor in the development of obesity-related complications, [21] with much higher lipolytic activity observed in visceral adipose tissue (VAT) than in subcutaneous adipose tissue (SAT). This characteristic is associated with increased expression and functional activity of β3-adrenoreceptors and fewer insulin receptors in visceral adipocytes, which leads to more intensive metabolism of lipids in VAT than in other fat depots [22]. The portal vein also passes through the VAT, which facilitates the entry of free fatty acids (FFA) into the liver. Excessive intake of FFA by hepatocytes leads to decreased insulin sensitivity and the development of insulin resistance (IR) and systemic hyperinsulinemia, which subsequently contributes to the development of peripheral IR [23]. Moreover, both IR and excess FFA levels lead to impaired lipid metabolism and the development of atherogenic dyslipidaemia [24]. In obese patients, adipocytes grow and accumulate triglycerides, which is accompanied by increased leptin expression and the development of leptin resistance [25]. Leptin resistance leads to increased FFA synthesis from de novo glucose because of the overexpression of numerous proteins that participate in this process, and this synthesis is independent of the plasma FFA concentration [26]. Moreover, an increased leptin concentration and decreased number of its receptors leads to the production of pro-inflammatory cytokines (e.g., TNF-α and IL-1) and blocks the production of anti-inflammatory cytokines (e.g., IL-4) [27]. Thus, leptin and inflammatory markers have a compounding relationship, with pro-inflammatory cytokines increasing the synthesis and release of leptin, which in turn helps maintain a chronic inflammatory condition in obese patients. When visceral obesity (VO) is combined with leptin resistance, leptin may induce vascular calcification, cholesterol accumulation by macrophages, oxidative stress, an increased tone of the sympathetic nervous system, and increased blood pressure [28]. All of these factors lead to decreased arterial compliance as a result of the atherosclerotic processes.

In 1997, Anderson et al. reported that a threshold of 132 cm^2^ for VAT area was associated with cardiovascular risk among patients with type 2 diabetes [29]. In addition, Després et al. and Sironi et al. have reported that VAT area is associated with an increased risk of CAD [30, 31], with a VAT area of > 131 cm^2^ being associated with increased coronary disease risk among men. Cardiologists at the Mayo Clinic have also found that the distribution of adipose tissue has the greatest effect on cardiovascular risk and mortality among patients with normal body weight, as VO in this population was associated with 2.75-fold higher cardiovascular risk and 2.08-fold higher risk of all-cause death than among people without VO [32]. In this context, the significant prevalence of VO (up to 40%) among patients with normal BMI and CAD is of great importance [33]. Moreover, Desprès and Lamarche revealed that pre- and post-menopausal women with a VAT area of > 110 cm^2^ had an increased risk of coronary heart disease [34].

One large study of patients with MS aimed to determine whether visceral fat could be assessed using absolute and/or relative quantitative indicators. The Japanese and Korean researchers evaluated the ratio of the areas for intra-abdominal VAT and SAT, and found that it was closely related to abnormal carbohydrate and fat metabolism in obese people, with these metabolic parameters being significantly higher in the “visceral” group (VAT/SAT of ≥0.4) than in the “subcutaneous” group (VAT/SAT of < 0.4) [35]. The same authors found that the abnormal carbohydrate and lipid metabolism in the “visceral” group was independent of sex, age, and BMI, although the VAT/SAT values increased with age among the general study population and was higher in men than in women. Therefore, it appears that VAT surrounding the internal organs is associated with cardiometabolic risk factors, regardless of total fat mass.

### Non-alcoholic fatty liver disease

Non-alcoholic fatty liver disease (NAFLD) is caused by hepatic steatosis (predominantly involving triglycerides) in individuals who do not consume sufficient alcohol quantities to exacerbate liver damage. The literature has repeatedly highlighted the interconnectivity of non-alcoholic steatohepatitis (NASH) and MS [36]. However, only a radiological examination can accurately quantify the liver’s fat content, with MRI and CT results revealing a direct relationship between steatosis severity and increased risks of type 2 diabetes and CVD [37]. These results can be explained by the fact that the liver is the key regulator of carbohydrate and lipid metabolism.

The pathogenesis of NASH is rooted in an imbalance between the synthesis and utilization of triglycerides and other cholesterol derivatives, which leads to excessive accumulation in hepatocytes. This condition is accompanied by increased lipolysis and very-low-density lipoprotein secretion, [38] which leads to atherogenic dyslipidaemia (elevated low-density and decreased high-density lipoprotein concentrations), [39] hyperglycaemia due to impaired insulin sensitivity and glucose hyperproduction, and the increased release of inflammatory factors, such as IL-6, TNF-α, and C-reactive protein [40]. These metabolic disorders can lead to atherosclerosis in patients with NASH, and a number of studies have demonstrated that NASH is associated with thickening of the carotid arteria complex and coronary atherosclerosis, [41] endothelial dysfunction, and coronary heart disease [40]. In addition, the RISC study revealed that excess fat accumulation in the liver was associated with increased coronary risk, even among patients who are thought to have low cardiovascular risk based on the absence of type 2 diabetes and hypertension [42]. Moreover, patients with NASH, even without MS, are more likely to have unstable coronary plaques than patients without NASH [43].

### Epicardial adipose tissue

Epicardial adipose tissue (EAT) is a multifaceted fat depot with unique local effects, systemic effects, anatomical characteristics, and metabolic properties. For example, relative to other fat depots, EAT has significantly higher FFA synthesis and increased FFA release in response to catecholamine stimulation. Intensive lipolysis in epicardial adipocytes may be associated with a low sensitivity to insulin and a large number of β3-adrenoreceptors [44]. In addition, EAT has higher protein content and lower glucose oxidation capacity than VAT, [45] as well as increased secretion of inflammatory factors (IL-1, IL-6, soluble IL-6 receptor, and TNF-α) in EAT relative to SAT [46]. Furthermore, the secretion of adipokines (adiponectin and adrenomedullin) that protect against CAD is approximately 40% lower in patients with EAT that in patients without CVD [46].

Under physiological conditions, epicardial adipocytes perform a number of functions that are important for the myocardium: metabolic (absorption of excess FFA and providing energy during ischemia), thermogenic (protection from overheating), mechanic, and textural (synthesizing adiponectin and adrenomedullin) [47]. However, in the context of obesity, the positive functions are replaced by negative functions, with the increased epicardial fat being accompanied by hypertrophy of the myocardium, fibrosis and apoptosis of cardiomyocytes, decreased synthesis of adiponectin, and increased production of inflammatory factors [48]. Thus, the balance between the protective and pathological effects of EAT is extremely fragile. Increases in EAT can lead to excess production of FFA, which prevents the generation and propagation of a nerve impulse through the heart fibres and subsequently potentiates the development of ventricular arrhythmias [49]. In contrast, the high lipolytic activity of EAT can generate the required energy for the myocardium during periods of ischemia. For example, patients with ischemic heart disease have significantly higher EAT expression and secretion of sPLA2-IIA (secretory phospholipase A2 II type) than patients without CAD, with sPLA2-IIA catalysing hydrolysis of phospholipids’ sn-2-ester bond with the formation of FFA and lysophospholipids. Nevertheless, it is unclear whether these changes are a cause of FFA hyperproduction during obesity, and further studies are needed to evaluate the participation of EAT in the pathogenesis of cardiovascular dysfunction.

Two large multi-ethnic studies (the Multi-Ethnic Study of Atherosclerosis and the Framingham Heart Study) have identified that fat deposits around the heart are an independent predictor of CVD risk [50]. In these studies, the thickness and volume of EAT was greater in patients with CAD than in control patients, as well as in patients with unstable angina relative to patients with stable angina or atypical chest pain. Interestingly, among patients with ischemic heart disease, EAT thickness is correlated with failure of the coronary bed, and autopsy data indicate that EAT volume is also correlated with myocardial hypertrophy [51]. Thus, in obese patients, EAT thickness is associated with the mass of the left ventricle’s myocardium and the size of the right ventricle’s cavity, while increased EAT in patients with a normal BMI is associated with more severe coronary artery lesions. Moreover, EAT thickness is significantly greater in patients with MS, [52] with EAT volume being directly correlated with some MS components, such as visceral obesity, fasting hyperglycaemia, myocardial infarction, hypertension, increased triglyceride concentrations, and decreased HDL concentrations [53]. Therefore, measuring EAT thickness is practically useful, as thickness or volume are directly correlated with visceral obesity, CAD, MS, and NASH, which indicates that EAT may accurately reflect cardiovascular risk and be useful for evaluating drugs that affect adipose tissue volume and endocrine function.

### Perivascular adipose tissue

Perivascular adipose tissue (PVAT) refers to fat clusters around vessels with various calibres. For example, the fatty tissue of the vascular network involving the heart, kidneys, mesentery, and muscles are a complete component of the vascular wall and is closely related to its other constituents, with no barriers separating PVAT from the adventitia [54]. This tissue includes a mixture of white and brown adipose tissues, with the precise ratio varying significantly according to the related blood vessel. Frontini et al. have reported that brown adipose tissue predominantly surrounds the aorta and its main branches (carotid, subclavian, intercostal, and renal arteries) [55]. Interestingly, Sacks et al. have reported that genetic markers indicate that the perivascular adipocytes surrounding the right coronary artery correspond to brown adipose tissue, [56] while Chatterjee et al. have reported that the gene expression profiles of perivascular adipocytes surrounding the coronary arteries correspond to white adipose tissue [57]. This may indicate that it is not always possible to separate the perivascular tissue from the epicardial fat depot, as there is no separating fascia, although it is also possible that different coronary arteries are covered with fat tissues of different origins. Other authors have attributed this phenomenon to the external environment, with lower temperatures promoting the development of brown adipose tissue and dietary restriction promoting the development of white adipose tissue, which is consistent with their functions in the body [58].

Measurement of the PVAT tissue thickness using CT revealed that the amount of PVAT is directly correlated with the VAT area and moderately correlated with the SAT area and body weight [59]. However, only a small number of studies have evaluated the effect of PVAT thickness on the development of insulin resistance. For example, one study revealed that PVAT thickness at the brachial artery was significantly correlated with insulin resistance [60]. Furthermore, in the Framingham Heart Study, thickness around the thoracic aorta was significantly correlated with BMI, VO, arterial hypertension, and type 2 diabetes mellitus [61].

The data presented above reveal variability in the effects of local fat depots on the risk of CVD development and progression, which can be explained by several factors. First, mammals have three phenotypes of fat tissue forming the depots (white, beige, and brown adipose tissue), which have different functions, phenotypes, anatomical localizations, morphology, origins, and development [62]. For example, white adipose tissue stores energy in the form of lipids that can be secreted for use in other tissues, and is located in the subcutaneous fat and surrounding the internal organs of the abdominal cavity. Brown adipose tissue is mainly located in the mediastinum, possesses unique thermogenic properties, and is a vital organ for maintaining a constant body temperature in small mammals and babies with a high surface area-to-volume ratio. Beige or brownish-white adipose tissue is predominantly found in white adipose tissue and develops a brown phenotype after prolonged cold exposure or pharmacological stimulation [63]. These three adipose tissue phenotypes have morphological differences and unique endocrine functions, which allows them to play important roles in human metabolism, especially in relation to obesity and its associated diseases, such as CVD.

An example of a phenotypic difference within a single depot is the para-aortic fatty tissue, with thoracic para-aortic fatty tissue being morphologically similar to brown adipose tissue and being comprised of adipocytes with a multi-coloured appearance and round nucleus. Direct comparison of murine PVAT gene expression in the thoracic aortic and intercapsular white and brown adipose tissues revealed significant differences in the expression of only 228 genes (i.e., 0.79%) between adipocytes in the thoracic aorta and classical brown adipose tissue. In contrast with thoracic aortic fatty tissue, abdominal aortic fatty tissue is more similar to white adipose tissue [64], especially in obese mice, where the abdominal aortic PVAT is similar to white adipose tissue (i.e., a large lipid compartment in each adipocyte). In addition, mesenteric PVAT is characterized by adipocytes with large lipid drops and low levels of uncoupling protein-1 expression.

Obesity can also be related to changing local fat depots, with excessive accumulation of subcutaneous fat being accompanied by an increase in the number of adipocytes and the absence of metabolic disorders. However, the accumulation of visceral fat leads to an increase in the size of adipocytes and increases their sensitivity to the effects of catecholamines, intense lipolysis, the development of insulin resistance, and adipokine and proinflammatory imbalance. In addition, visceral adipocytes (unlike subcutaneous adipocytes) are characterized by a high density of androgenic corticosteroid receptors, rich innervation, a wide capillary network, and a high metabolic activity. Thus, prostate tissue adipocytes predominantly exhibit adiponectin production, whereas SAT adipocytes predominantly synthesize leptin. Epicardial adipocytes have high proinflammatory activity, whereas most perivascular adipocytes do not synthesize TNF-alpha [65]. These differences may be related to the phenotypes of the different adipose tissues. For example, the unfavourable metabolic effects of VAT are facilitated by anatomical proximity to the portal vein, which passes through the abdominal fat and allows factors that are formed during FFA lipolysis to reach the liver. Excess hepatic intake of FFA leads to decreased sensitivity to insulin and the development of insulin resistance and systemic hyperinsulinemia, which increase the production of “hepatic” glucose. In hypertrophied adipocytes, the insulin-dependent glucose uptake is reduced due to deficiency of the GLUT4 receptors, which aggravates hyperglycaemia and insulin resistance. In addition, systematic circulation of FFA contributes to decreased glucose uptake and its utilization in muscle tissue, which strengthens peripheral insulin resistance. Excess FFA and insulin resistance, combined with visceral obesity, lead to disruption of lipid metabolism and the development of atherogenic dyslipidaemia. Thus, disruption of carbohydrate and lipid metabolism creates a vicious cycle, with FFA synthesized by visceral adipocytes serving as a key catalyst. Therefore, each local fat depot can be considered an independent endocrine organ that actively produces biologically active molecules, such as pro- and anti-inflammatory cytokines and adipokines, although the effects of each depot vary greatly.

## Conclusion

**Fig. 1:**
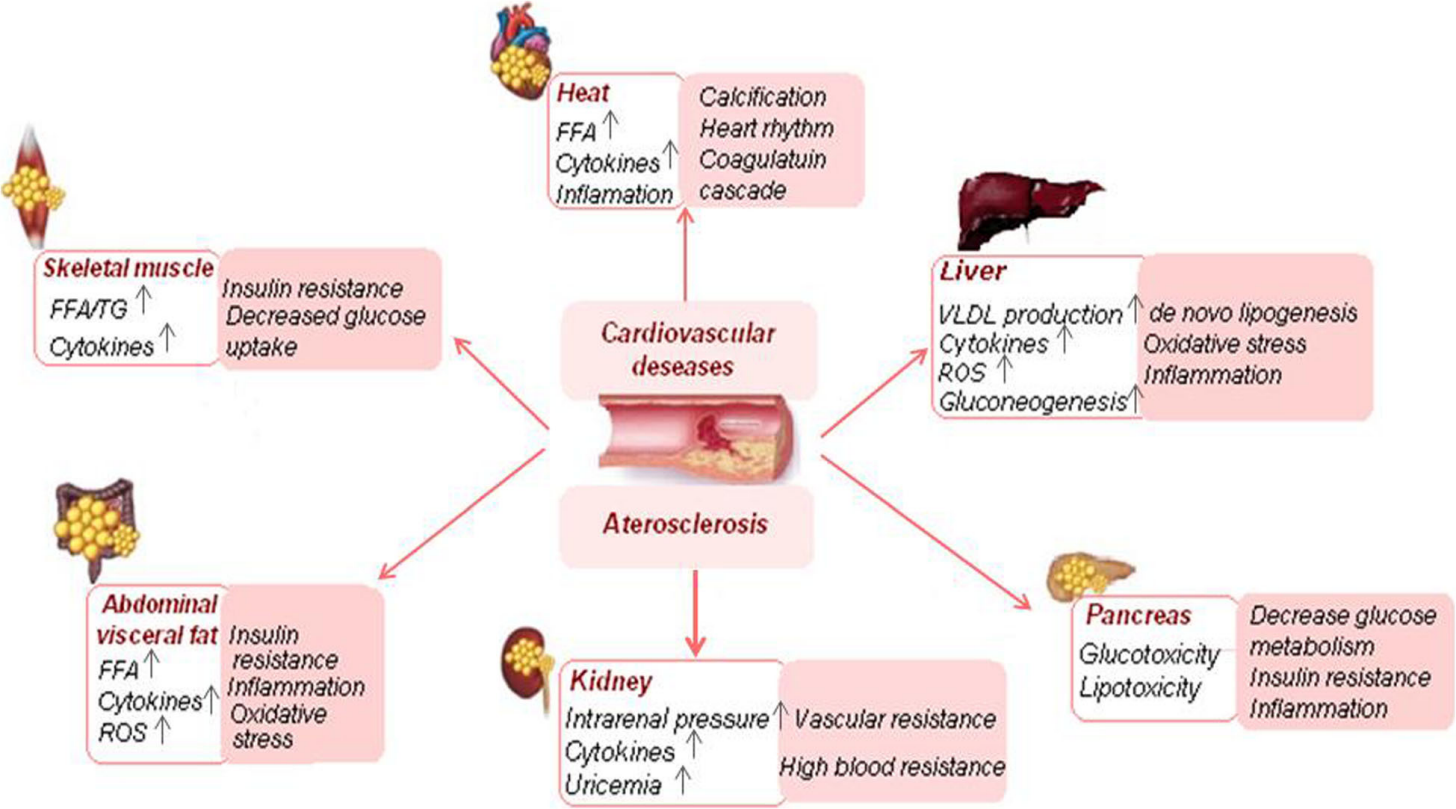
Mechanisms of various ectopic fats related with cardiovascular deseases

It is known that each local fat depot can be considered an independent endocrine organ that actively produces biologically active molecules, such as pro- and anti-inflammatory cytokines and adipokines. However, the effects of each depot vary greatly, with SAT adipocytes predominantly producing adiponectin and VAT adipocytes more actively synthesizing leptin. Epicardial adipocytes have a high pro-inflammatory activity, whereas most perivascular adipocytes do not synthesize TNF-alpha. These mechanistic differences may be attributed to the phenotype of each adipose tissue depot. For example, the properties of PVAT may be attributed to brown adipose tissue, including its cellular morphology and the expression of characteristic thermogenic genes. However, the phenotype of PVAT near other vessels is relatively heterogeneous, which may be attributed to the phylogenetic origins of PVAT and other adipose tissues. Thus, it remains unclear whether PVAT is a classic brown, beige, or white adipose tissue with changing characteristics, and similar phenotypic properties are manifested by paranephric fatty tissue. Accumulating evidence suggests that the regional distribution of adipose tissue plays an important role in the development of MS and CVD (Fig. 1). Although most ectopic fat depots are interrelated, future cardiology studies would help increase our understanding of their involvement in the pathophysiological mechanisms of CVD development, such as stenocardia, myocardial infarction, atrial fibrillation, heart failure, stroke, and aortic stenosis.

## Abbreviations

BMI: Body mass index
CAD: Coronary artery disease
CT: Computed tomography
CVD: Cardiovascular diseases
EAT: Epicardial adipose tissue
FFA: Free fatty acids
IR: Insulin resistance
MS: Metabolic syndrome
NAFLD: Non-alcoholic fatty liver disease
NASH: Alcoholic steatohepatitis
PVAT: Perivascular adipose tissue
SAT: Subcutaneous adipose tissue
VAT: Visceral adipose tissue
VO: Visceral obesity
